# Dual Transradial Approach for Aortoiliac Occlusive Disease

**DOI:** 10.1016/j.jaccas.2025.104410

**Published:** 2025-07-30

**Authors:** Yoshimitsu Soga, Kento Matsui, Kenji Ando

**Affiliations:** Department of Cardiology, Kokura Memorial Hospital, Kitakyushu, Japan

**Keywords:** aorta, peripheral vascular disease, stents

## Abstract

**Background:**

The effectiveness of the dual transradial approach (TRA) for aortoiliac occlusive disease (AIOD) remains unclear.

**Case Summary:**

A 62-year-old man presented with claudication in his both legs. Bilateral ankle-brachial index was decreased, and computed tomography demonstrated the aortoiliac occlusion. Because medical therapy was insufficient, revascularization was performed. A guiding sheath was inserted from the left radial artery to the distal aorta, and 2 guidewires were passed through both common iliac arteries (CIAs). After the balloon angioplasty for each CIA, new guiding sheath was additionally inserted from the right radial artery. A guidewire was advanced into the right CIA. Finally, a bare-nitinol stent was placed in each CIA from both radial arteries with no complications.

**Discussion:**

Although the bilateral or unilateral transfemoral approach was conventionally selected for AIOD, dual TRA could be one of the options.

**Take-Home Message:**

Dual TRA could be considered as an alternative treatment for AIOD.

## Visual Summary

**Figure:**
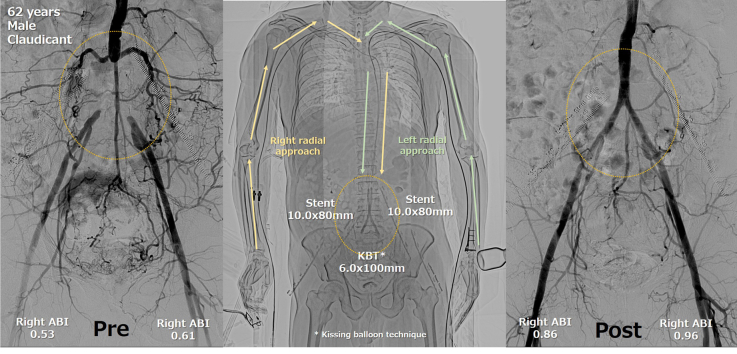

**Visual Summary:**
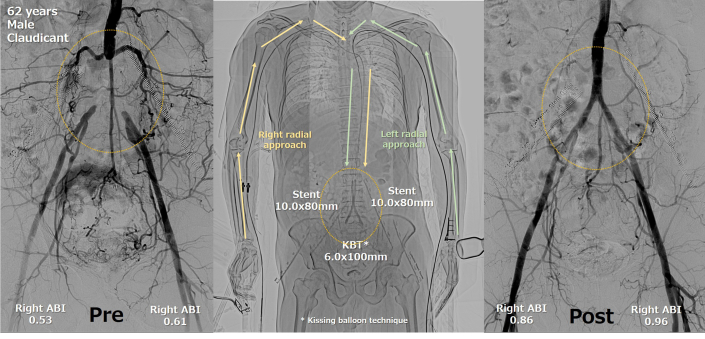
Dual Transradial Intervention for Aortoiliac Occlusion

Endovascular treatment for aortoiliac occlusive disease (AIOD) has been reported.^1^, ^2^, ^3^ However, most of the reports are based on the transfemoral approach, and the transradial approach (TRA) alone for AIOD remains unclear. We report AIOD treated with dual TRA.

**Take-Home Messages**

• Although a complex lesion such as aortoiliac occlusive disease has been generally treated with the transfemoral approach, the procedure could be completed using a dual transradial approach.
• It added a procedural option and could be an alternative treatment for aortoiliac occlusive disease.

## History of Presentation

A 62-year-old man presented with intermittent severe claudication (Rutherford category 3) in his both legs for several months. He was diagnosed with lower extremity artery disease based on a decreased bilateral ankle-brachial index (ABI). Because his claudication did not improve despite medical therapy, he was referred to our hospital for revascularization.

## Past Medical History

His past medical history was hypertension and paroxysmal atrial fibrillation, which were treated with antihypertensive drug and anticoagulant.

## Differential Diagnosis

Thromboembolism caused by atrial fibrillation, lumber canal stenosis, and lumbar spondylosis are the differential diagnoses. Given the calcified lesion based on computed tomography (CT), it was less likely thromboembolism. Other potential causes, such as lumber canal stenosis or lumbar spondylosis, were less consistent with the imaging and clinical findings.

## Investigations

**Figure 1:**
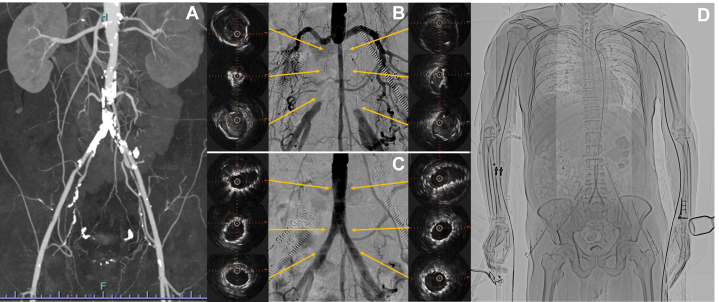
Overview of Dual Transradial Intervention for Aortoiliac Occlusion (A) CT angiography, (B) preprocedural angiography and IVUS findings after guidewire passage, (C) postprocedural angiography and final IVUS findings, and (D) dual transradial approach. CT = computed tomography; IVUS = intravascular ultrasound.

ABI was decreased bilaterally (right 0.53, left 0.61). Duplex ultrasound showed occlusion from the distal aorta to the common iliac artery (CIA). Good flow was observed in the bilateral radial arteries. Contrast-enhanced CT showed occlusion with severe calcification from the distal aorta to the CIA (Figure 1A). No stenosis and fragile plaque were observed in other arteries.

## Management (Intervention)

A 6-F guiding sheath (BRITE TIP RADIANZ 110 cm, Cordis) was initially inserted through the left radial artery to the distal aorta, and angiography showed the aortoiliac occlusion (Video 1). First, a 0.014-inch guidewire (Gladius 300 cm, Asahi Intec) with a microcatheter (TORQ Porter NEO 135 cm; Tokai Medical Products) was manipulated and advanced through the right CIA; then another guidewire (Astato9-40 300 cm; Asahi Intec) with the previous microcatheter was also advanced through the left CIA, and intraluminal crossing in both CIAs was confirmed by intravascular ultrasound (Figure 1B, Videos 2 and 3). Each CIA was alternately dilated with a balloon (Oceanus 14 RX 6.0 × 100 mm, iVascular, Video 4). After dilatation, a new 6-F guiding sheath (BRITE TIP RADIANZ 110 cm; Cordis) was additionally inserted from the right radial artery to the distal aorta. A guidewire (Gladius 300 cm; Asahi Intec) was then inserted into the right CIA, which was dilated with balloon, and the previous guidewire (Astato9-40) from the left radial approach was removed. A 10.0 × 80 mm bare-nitinol stent (SMART RADIANZ; Cordis) was placed in each CIA from both radial sheath, and the kissing balloon technique was performed with the same 6.0 × 100 mm balloons (Oceanus 14 RX 6.0 × 100 mm, iVascular). Final angiography showed good blood flow without any complications (Video 5), and intravascular ultrasound confirmed good stent expansion (Figure 1C, Videos 6 and 7).

## Outcome and Follow-Up

The patient was ambulatory immediately after the procedure and did not require bed rest. Postoperative ABI improved to 0.86 on the right and 0.96 on the left, and subjective symptoms disappeared. Each radial artery was palpable and patent by duplex.

## Discussion

In this case, the occlusion area was highly calcified on CT, but there was mainly circumferential calcification and partially protruding calcified nodule. The CT values of the occlusion were relatively low, and the plaque was considered soft. The antegrade guidewire passage was expected to be easy. To avoid distal embolization, vessel-size predilation was not needed, and primary stenting was performed. The covered stent was not selected because of the concerns of main-branch occlusion by protrusions, and the covered endovascular reconstruction of aortic bifurcation^4^^,^^5^ technique was not considered because of the short aortic occlusion length, and a kissing stent with a self-expanding stent was preferred over a balloon-expandable stent because of the difference diameter between the aorta and CIA and considering stent apposition in the chronic phase. Although radial access for peripheral intervention is gaining popularity, there are some limitations. First, retrograde access is necessary in some cases. In this situation, only microcatheter use as retrograde femoral access can reduce the invasiveness. Second, there are specific complications of TRA such as radial artery occlusion and cerebral infarction. In addition, it may be difficult to deal with serious complication when it occurs. Third, the length and type of devices are limited. Finally, radiation exposure may be high, and radiation protection measures (shield or positioning) are necessary.

## Conclusions

We treated AIOD with dual TRA (Figure 1D). This approach could be an alternative to the transfemoral approach for patients with AIOD.

## Funding Support and Author Disclosures

The authors have reported that they have no relationships relevant to the contents of this paper to disclose.

## Appendix

Video 1 Preprocedural Angiogram

Video 2 Preprocedural Intravascular Ultrasound From the Distal Aorta to the Left Common Iliac Artery

Video 3 Preprocedural Intravascular Ultrasound From the Distal Aorta to the Right Common Iliac Artery

Video 4 Angiogram After Balloon Angioplasty

Video 5 Final Angiogram

Video 6 Final Intravascular Ultrasound From the Distal Aorta to the Right Common Iliac Artery

Video 7 Preprocedural Intravascular Ultrasound From the Distal Aorta to the Right Common Iliac Artery
